# Co-Doping Effect of Mn^2+^ and Eu^3+^ on Luminescence in Strontiowhitlockite Phosphors

**DOI:** 10.3390/molecules29010124

**Published:** 2023-12-24

**Authors:** Ivan V. Nikiforov, Dmitry A. Spassky, Nataliya R. Krutyak, Roman Yu. Shendrik, Evgenia S. Zhukovskaya, Sergey M. Aksenov, Dina V. Deyneko

**Affiliations:** 1 Department of Chemistry, Lomonosov Moscow State University, 119991 Moscow, Russia; 2 Skobeltsyn Institute of Nuclear Physics, Lomonosov Moscow State University, 119991 Moscow, Russia; 3 Institute of Physics, University of Tartu, 50411 Tartu, Estonia; 4 Vinogradov Institute of Geochemistry, Siberian Branch of the Russian Academy of Sciences, 664033 Irkutsk, Russia; 5 Laboratory of Arctic Mineralogy and Material Sciences, Kola Science Centre, Russian Academy of Sciences, 184209 Apatity, Russia; 6 Geological Institute, Kola Science Centre, Russian Academy of Sciences, 184209 Apatity, Russia

**Keywords:** β-Ca_3_(PO_4_)_2_, β-TCP, strontiowhitlockite, phosphors, phosphate, Mn^2+^/Eu^3+^, luminescence, LED

## Abstract

A new series of Sr-based phosphates, Sr_9−*x*_Mn*_x_*Eu(PO_4_)_7_, were synthesized using the high-temperature solid-state method in air. It was found that these compounds have the same structure as strontiowhitlockite, which is a β-Ca_3_(PO_4_)_2_ (or β-TCP) structure. The concentration of Mn^2+^ ions required to form a pure strontiowhitlockite phase was determined. An unusual partial reduction of Eu^3+^ to Eu^2+^ in air was observed and confirmed by photoluminescence (PL) and electron spin resonance (ESR) spectra measurements. The PL spectra recorded under 370 nm excitation showed transitions of both 4*f*5*d*–4*f* Eu^2+^ and 4*f*–4*f* Eu^3+^. The total integral intensity of the PL spectra, monitored at 395 nm, decreased with increasing Mn^2+^ concentration due to quenching effect of Eu^3+^ by the Mn^2+^ levels. The temperature dependence of Eu^2+^ photoluminescence in a Sr_9−*x*_Mn*_x_*Eu(PO_4_)_7_ host was investigated. The conditions for the reduction of Eu^3+^ to Eu^2+^ in air were discussed.

## 1. Introduction

One of the primary objectives in addressing light-emitting diode (LED) issues is to identify an optimal host for harnessing the photoluminescence properties of cation activators. Another objective is to regulate emissions through chemical deposition. Research has clearly shown that certain phosphates [1,2], aluminates [3,4], silicates [5,6], glasses [7,8], and so on serve as excellent hosts for rare-earth elements and transition metals with photoluminescence properties in the visible region. However, each of these hosts has its drawbacks, such as a high synthesis temperature with reduced atmosphere [9], non-green chemistry preparation techniques [10], low isomorphic capacity, and variation in the substitution types [11]. In this context, β-Ca_3_(PO_4_)_2_-type (or β-TCP) phosphors are considered suitable materials due to their wide capacity for substitutions of Ca^2+^ ions with either homovalent or heterovalent ions, resulting in excellent properties [12].

Calcium-based phosphates with a β-TCP structure are still of great interest. However, it has been shown that Sr-based phosphates with a β-Ca_3_(PO_4_)_2_ structure exhibit photoluminescence properties that are several times higher [13,14]. The related mineral—strontiowhitlockite [15], or Sr_9_Mg(PO_4_)_6_(PO_3_OH)—is the strontium analogue of whitlockite or β-Ca_3_(PO_4_)_2_ and also belongs to the cerite supergroup. The replacement of Ca^2+^ with Sr^2+^ ions leads to an increase in the photoluminescence properties [16] or their modification [17,18] due to the formation of more distorted luminescence center environments. It is worth noting that pure Sr_3_(PO_4_)_2_ differs from the β-Ca_3_(PO_4_)_2_ structure [19] and is known as a mineral palmierite family member. Furthermore, a comprehensive substitution of Ca^2+^ → Sr^2+^ was investigated in detail in [20]. It was shown that Sr-based phosphates form a β-Ca_3_(PO_4_)_2_ structure only with the stoichiometric formula Sr_8_*M*^2+^*R*^3+^(PO_4_)_7_ [21] or Sr_9_*R*´^3+^(PO_4_)_7_ (where *R*^3^ is a rare earth element, and *R*´^3+^ is a tripositive ion, such as Sc^3+^ or Fe^3+^, [2,22]). Despite numerous articles on the photoluminescence properties of strontiowhitlockite-type phosphors, the full concentration series with Sr^2+^ → *M*^2+^ or Sr^2+^ → *R*^3+^ has not been discussed. However, a similar series on Ca-based phosphates, such as Ca_9−*x*_*M*^2+^*_x_*Eu(PO_4_)_7_, was previously described in detail for *M*^2+^ = Zn^2+^ [23], Mg^2+^ [24], and Mn^2+^ [25]. 

The control of luminescence properties is achieved by using different pairs of ions in the host material, such as Tb^3+^/Eu^3+^ [26], Sm^3+^/Eu^3+^ [27], Ce^3+^/Mn^2+^ [28], Ce^3+^/Tb^3+^ [29], Eu^2+^/ Mn^2+^ [30], and others. The choice of these pairs is based on the existence of energy transfer processes between them, resulting in unique combinations of emitting colors. Another method to modify photoluminescence is by changing the oxidation state [31,32] of the ion-activator. 

In this study, we investigate Sr-based phosphors with the β-TCP structure and common formula Sr_9−*x*_Mn*_x_*Eu(PO_4_)_7_. The influence of Mn^2+^ and Eu^3+^ co-doping on the photoluminescence properties as well as the impact of homovalent Ca^2+^ → Mn^2+^ substitution on the structure formation are also investigated. The abnormal self-reduction process of Eu^3+^ ions in the strontiowhitlockite host in air was observed.

## 2. Results and Discussion

### 2.1. PXRD and SHG Study

**Figure 1:**
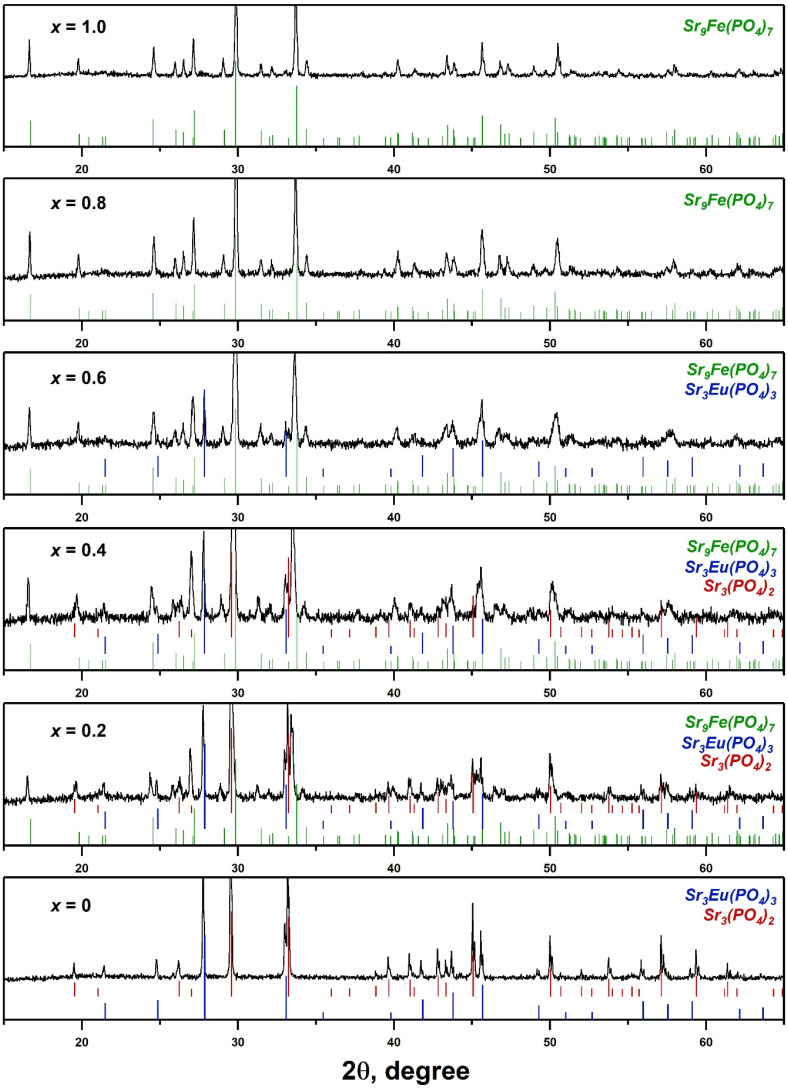
The PXRD patterns of Sr_9−*x*_Mn*_x_*Eu(PO_4_)_7_ and the Bragg reflections of Sr_9_Fe_1.5_(PO_4_)_7_ (PDF-2 Card 51–427), Sr_3_Eu(PO_4_)_3_ (PDF–2 Card 48–410), and Sr_3_(PO_4_)_2_ (PDF–2 Card 85–502).

The PXRD patterns of SrMn*x*Eu are shown in Figure 1. Unlike the similar Ca-based solid solution Ca_9−*x*_Mn*_x_*Eu(PO_4_)_7_ [25], an unbroken series of solid solutions with the strontiowhitlockite structure was not formed. However, the Sr_8_MnEu(PO_4_)_7_ sample crystallized in the trigonal Sr_9_Fe_1.5_(PO_4_)_7_-type, or strontiowhitlockite-type, structure (space group (sp.gr.) *R*$\bar{3}$*m*, PDF-2 Card 51–427) (Figure 1). It appears that Mn^2+^ ions in the octahedra site played a critical role in the structure stabilization, similar to the Mg^2+^ ions in strontiowhitlockite. A similar structure formation was found in some related works, for example for Sr_9_MnK(PO_4_)_7_:Eu^2+^,Ce^3+^ [33] or Sr_8_MgCe(PO_4_)_7_:Eu^2+^,Mn^2+^ [34] on Sr-based phosphors.

Among the synthesized compounds, only the SrMn0.8Eu and SrMn1.0Eu samples were single-phased. Therefore, the limit content of Mn^2+^ ions required to form the strontiowhitlockite structure is *x* = 0.8. The observed reflections on the PXRD patterns for SrMn0Eu corresponded to the superposition of eulytite-typeSr_3_Eu(PO_4_)_3_ (sp.gr. *I*$\bar{4}$3*d*, PDF-2 Card 48–410) and palmierite-type Sr_3_(PO_4_)_2_ (sp.gr. *R*$\bar{3}$*m*, PDF-2 Card 85–502) structures. The samples with *x* = 0.2–0.4 were characterized by mixtures of phases with Sr_9_Fe_1.5_(PO_4_)_7_, Sr_3_(PO_4_)_2_, and Sr_3_Eu(PO_4_)_3_ structures (Figure 1). The quantitative analysis of the phase content calculated using the Jana2006 software is shown in Table 1. 

The formation of the β–Ca_3_(PO_4_)_2_-type structure has been described in previous studies [35,36]. These studies found that that samples with the general stoichiometric formula Sr_9_*R*(PO_4_)_7_ (where *R* = La–Sm) did not crystallize in the strontiowhitlockite structure. However, the Ca-based phosphate series with the general formula Ca_9_*R*^3+^(PO_4_)_7_ is known to crystallize in the β-Ca_3_(PO_4_)_2_ structure [37]. 

**Figure 2:**
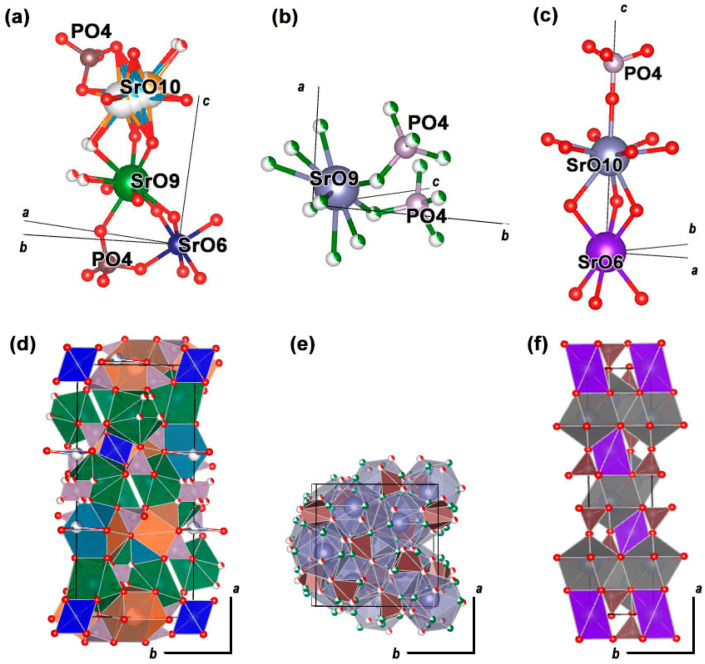
Oxygen environment of Sr^2+^ ions oriented along the *abc* axes and *ab* projection of structures, respectively: Sr_9_Fe_1.5_(PO_4_)_7_ (**a**,**d**), Sr_3_Eu(PO_4_)_3_ (**b**,**e**), and Sr_3_(PO_4_)_2_ (**c**,**f**).

The ionic radius of Sr^2+^ is significantly larger than that of Ca^2+^, resulting in structural defects that distort the β-Ca_3_(PO_4_)_2_ structure. This distortion can lead to the formation of eulytite-type Sr_3_*R*(PO_4_)_3_ phosphate, which contains an excess of Sr^2+^ ions. To stabilize the β-Ca_3_(PO_4_)_2_ structure in Sr-based phosphates, small ions such as Mn^2+^, Zn^2+^, Mg^2+^ can be added [13,38]. Figure 2 shows the different structural sites. In the case of Sr^2+^ with Eu^3+^, they occupy the largest sites as SrO_10_ and SrO_9_. The smallest Mn^2+^ ion prefers to occupy the octahedral SrO_6_ site in Sr_9_Fe_1.5_(PO_4_).

The presence of the non-centrosymmetric eulytite-type Sr_3_Eu(PO_4_)_3_ phase was confirmed through an SHG study. The SHG signal was dependent on the phase composition, which was determined using PXRD data. Consequently, the highest SHG signal was observed for SrMn0Eu, indicating a significant amount of the non-centrosymmetric eulytite-type phase. Increasing the concentration of Mn^2+^ in the SrMn*x*Eu solid solution resulted in a reduction in the eulytite-type phase, which was also evident in the decrease in the SHG signal. For the SrMn0.8Eu and SrMn1.0Eu samples with the strontiowhitlockite structure, the SHG signals were comparable to the systematic errors in the measurement method. 

### 2.2. ESR Analyze

**Figure 3:**
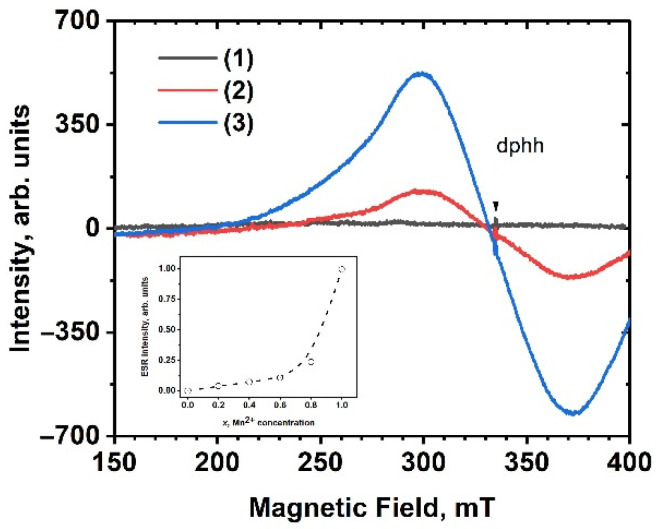
ESR spectra of Sr_9−*x*_Mn*_x_*Eu(PO_4_)_7_ *x* = 0 (1), *x* = 0.8 (2), and *x* = 1.0 (3) samples. The inset shows concentration dependence of integral intensity of ESR signal.

No ESR signal was detected in the sample without Mn, i.e., SrMn0Eu, while the Mn^2+^-containing samples showed a wide, structureless signal with a *g-*factor of 1.997 (Figure 3). The shape of the ESR spectra remained unchanged as the temperature cooled down to 77 K. Furthermore, the signal intensity displayed non-linear behavior in relation to the Mn concentration (Figure 3, inset).

On one hand, the observed ESR signal was attributed to the presence of manganese ions. This is supported by the fact that the ESR of Mn^2+^ exhibited a signal in a region with a *g*-factor close to 2 [39,40,41,42]. However, the characteristic sextet pattern of Mn^2+^ was not observed in the studied samples.

Similar ESR spectra are often observed in Eu^2+^-doped compounds [43,44,45]. This observation is further supported by the non-linear increase in the ESR signal intensity with the increase in the Mn^2+^ concentration, particularly in the samples with *x* = 0.8 and 1.0 (Figure 3 inset). Simultaneously, the luminescence of Eu^3+^ was quenched (see below). These finding suggest the presence of Eu^2+^ ions in the samples. The broadening of the ESR signal may be attributed to the exchange interaction between manganese and europium ions, similar to what has been observed in silicates [45]. 

### 2.3. Diffuse Absorption

**Figure 4:**
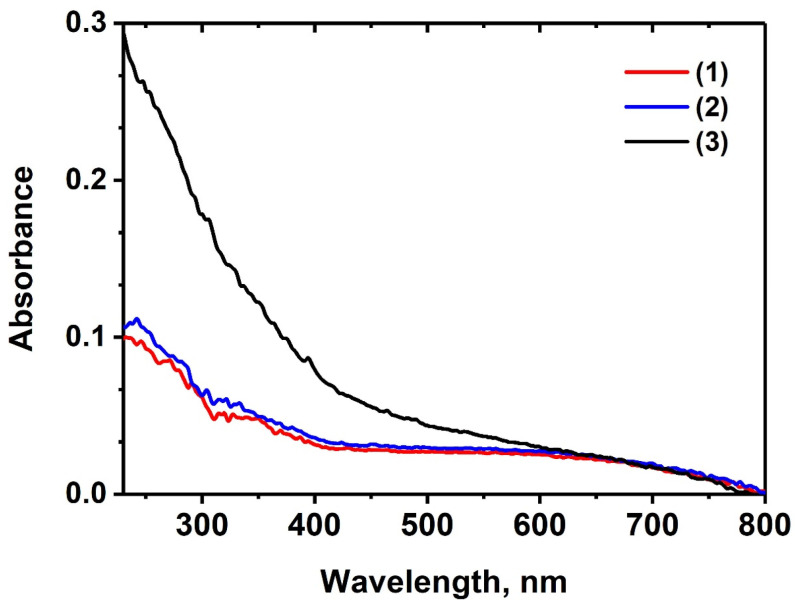
The diffuse absorption spectra of Sr_9−*x*_Mn*_x_*Eu(PO_4_)_7_: *x* = 0 (1), *x* = 0.6 (2), and *x* = 1.0 (3).

The diffuse absorption spectra of SrMn*x*Eu are shown in Figure 4. The spectra for the samples with *x* = 0 and 0.6 exhibited a similar structure. However, the absorption spectrum of the SrMn1.0Eu showed a prominent edge starting at 400 nm and extending towards the shorter-wavelength region of the spectrum (Figure 4). This behavior can be explained by the formation of a single-phase sample for SrMn1.0Eu. The absorption bands of Mn^2+^ are typically found in the 370–440 nm spectral range and are attributed to d-d transitions. These absorption bands often have a low oscillator strength and can appear broadened when Mn^2+^ ions occupy multiple non-equivalent positions within the lattice [46,47]. Additionally, a prominent absorption edge is observed in this region. The combination of these factors can mask the absorption bands of Mn^2+^.

It is possible that manganese ions can exist in a different oxidation state. One hypothetical substitution scheme could be Mn^2+^ + Eu^3+^ ↔ Mn^3+^ + Eu^2+^. An indicator of the presence of Mn^3+^ ions in the lattice is the occurrence of a broad absorption band in the visible spectral region, typically peaking around 450–700 nm [48]. The broadening of this band can be attributed to the occupancy of different positions by Mn^3+^ ions. A wide plateau of low intensity can be observed in the visible region of the presented spectra (Figure 4). However, it is difficult to confidently conclude the presence of manganese 3+ solely based on the absorption spectra.

### 2.4. Photoluminescence Properties 

**Figure 5:**
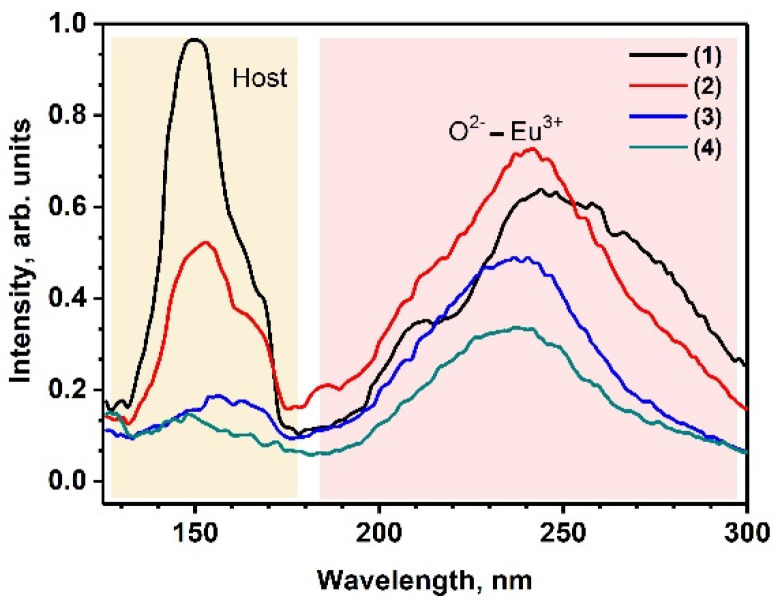
The excitation spectra of Eu^3+^ luminescence monitored at ^5^D_0_ → ^7^F_2_ emission band for Sr_9−*x*_Mn*_x_*Eu(PO_4_)_7_ *x* = 0 (1), *x* = 0.2 (2), *x* = 0.6 (3), and *x* = 0.8 (4).

The VUV excitation spectra of the Eu^3+^ 4*f–*4*f* luminescence, measured at the ^5^D_0_ → ^7^F_2_ band, are shown in Figure 5. In the SrMn0Eu sample undoped by Mn^2+^ ions, a broad band centered around 250 nm and a relatively sharp band around 150 nm were observed. The broad band at 250 nm was attributed to the charge transfer band from (CTB) O^2−^ to Eu^3+^, while the sharp band at 150 nm corresponded to host excitation. In the SrMn*x*Eu solid solutions, the position of the band at 250 nm shifted to a shorter wavelength as the concentration of Mn^2+^ ions increased. Additionally, the intensity of the band at 150 nm significantly decreased. At 120–160 nm, Mn^2+^ ions typically exhibit intra-ionic 3d-4s transitions [49,50], which have a high oscillator strength. This indicates strong absorption bands in this wavelength range. Consequently, the strong absorption leads to non-radiative relaxation of excitations, resulting in the quenching of luminescence when excited in this specific region.

**Figure 6:**
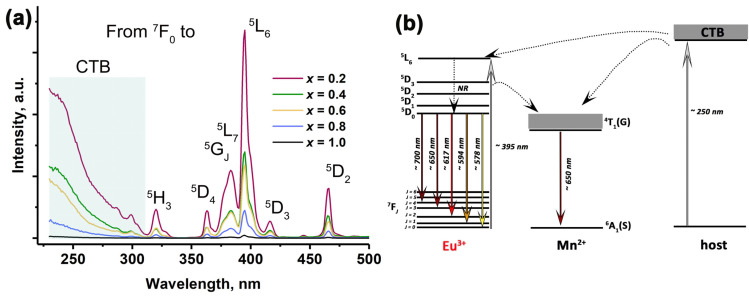
(**a**) The PLE spectra for Sr_9−*x*_Mn*_x_*Eu(PO_4_)_7_, monitored at 620 nm; (**b**) the proposed schema of energy transfer processes in host.

The photoluminescence properties of SrMn*x*Eu solid solutions are sensitive to phase purity and chemical composition. Figure 6a shows the PLE spectra. The number and position of the observed bands, corresponding to 4*f*–4*f* transitions of the Eu^3+^ ions, remained unchanged for the samples with *x* = 0.2–0.8. The broad band ranging from 250 to 300 nm was attributed to the CTB, while the sharp peaks in the range of 300–500 nm arose from the *f–f* transitions of Eu^3+^. Specifically, the peaks located at 320, 361, 376, 382, 395, 416 and 465 nm corresponded to the ^7^F_0_ → ^5^H_3_, ^5^D_4_, ^5^G_J_, ^5^L_7_, ^5^L_6_, ^5^D_3_, and ^5^D_2_ transitions of Eu^3+^ ions [5,51,52,53]. All the spectral lines in the SrMn*x*Eu host appeared to be wider when compared to those in other hosts that have been described. This could potentially be attributed to the presence of Eu^3+^ ions in the different environments. The presence of Mn^2+^ ions caused a notable reduction in the intensity of both the CTB and the standard transitions of Eu^3+^. The observed decrease in intensity was attributed to the increase in the Mn^2+^ concentration in the SrMn*x*Eu solid solutions. This decrease could be attributed to the quenching effect by Mn^2+^ and the abnormal reduction of Eu^3+^ during synthesis. The proposed energy transfer schema is shown in Figure 6b, with the most intensive line observed at 395 nm. 

**Figure 7:**
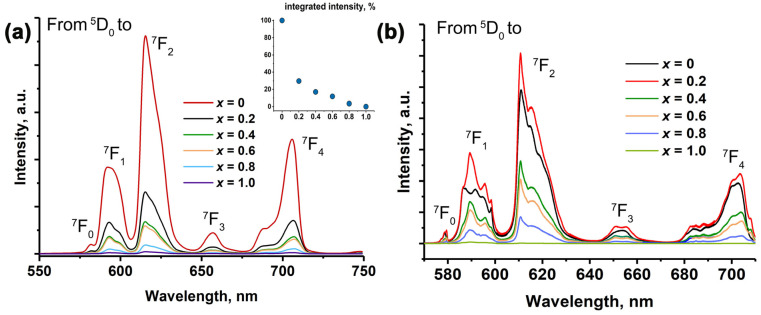
(**a**) The low-resolution and (**b**) high-resolution PL of Sr_9−*x*_Mn*_x_*Eu(PO_4_)_7_ (λ_exc_ = 395 nm) (the insert shows the integral intensity of the PL spectra on Mn^2+^ ion concentration).

The PL spectra for SrMn*x*Eu, excited at 395 nm (Figure 7), exhibited characteristic lines corresponding to Eu^3+^ transitions. The sharp lines at 578, 594, 617, 650, and 700 nm corresponded to the transitions ^5^D_0_ → ^7^F*_J_* (*J* = 0, 1, 2, 3, 4), with the main band at 615 nm. The resulting emission was observed in the red region of the visible spectrum [54,55]. Previous studies have shown that the total integral intensity is higher for the host Sr_8_*M*Eu^3+^(PO_4_)_7_ (where *M* = Mg, Zn) compared to Ca-based phosphates [13], regardless of the synthesis method. In this work, an increase in the Mn^2+^ concentration led to a decrease in the total integral intensity. Additionally, a gap was observed for the samples with *x* = 0 and 0.2 (Figure 7a, insert). 

A decrease in the total integral intensity of Eu^3+^ transitions was also observed for the single-phased SrMn0.8Eu and SrMn1.0Eu with a β-Ca_3_(PO_4_)_2_-type (or strontiowhitlockite) structure. This can be explained by the energy transfer process from the Eu^3+^ to Mn^2+^ levels through nonradiative transitions to the excited ^4^T_1_(G) state and emission to the ground ^6^A_1_(S) state. It is possible that the Mn^2+^ emission overlapped with the ^5^D_0_ → ^7^F_0,1,3_ Eu^3+^ transition [56,57], which can be observed in the high-spectral-resolution PL spectra. Furthermore, the emission of Mn^2+^ could be decreased through a concentration-quenching process. A proposed schema of this process is shown in Figure 7b. Similar behavior in the quenching of Eu^3+^ photoluminescence by Mn^2+^ ions has been previously observed in Ca_9−*x*_Mn*_x_*Eu(PO_4_)_7_ [25] and in isostructural Ca_3_(VO_4_)_2_:Eu^3+^, Mn^2+^ [58].

It is important to note that the spectral profile of the SrMn0Eu was significantly different compared to the others, as clearly seen in the high-spectral-resolution spectra (Figure 7b). The profiles for SrMn0.2Eu, SrMn0.4Eu, and SrMn0.6Eu were similar, indicating a similar oxygen environment. These samples consisted of two phases with β-TCP and eulytite types. This suggests that with the Mn^2+^ concentration at *x* = 0.2, Eu^3+^ primarily occupied sites in the whitlockite Sr_9_Fe_1.5_(PO_4_)_7_ and eulytite Sr_3_Eu(PO_4_)_3_ structures with an excess of Sr^2+^ ions forming the Sr_3_(PO_4_)_2_ phase. 

**Figure 8:**
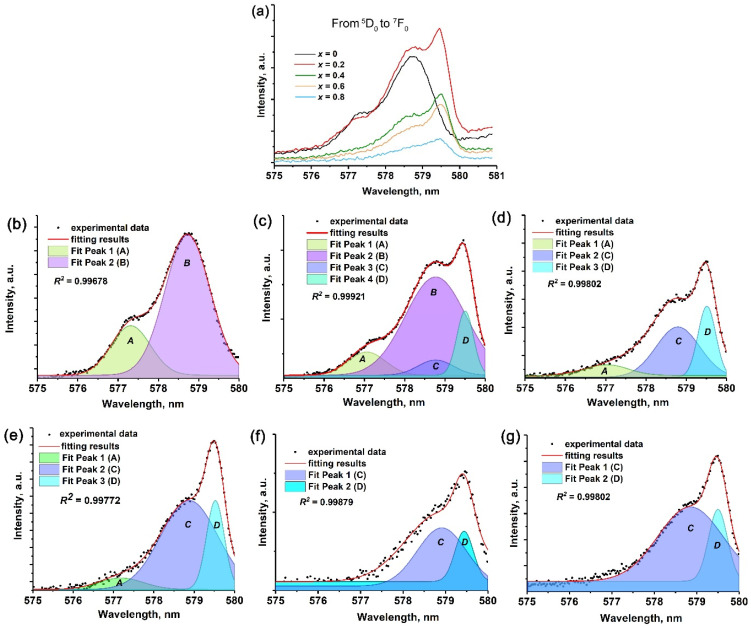
(**a**) The ^5^D_0_ → ^7^F_0_ transition for Sr_9−*x*_Mn*_x_*Eu(PO_4_)_7_, (**b**–**g**) fitting by Gauss components for *x* = 0, 0.2, 0.4, 0.6, 0.8, and 1.0, respectively, λ_exc_ = 395 nm.

Additional information about the phase composition and oxygen environment of the emission centers can be obtained thought consideration the forbidden electric dipole ^5^D_0_ → ^7^F_0_ transition of Eu^3+^ (Figure 8) [59]. The non-degenerate energy levels indicated the number of nonequivalent sites for Eu^3+^. For the sample with *x* = 0, this transition can be represented by two Gaussian components, indicating two non-equal oxygen environments for Eu^3+^. It should be noted that the Sr_3_(PO_4_)_2_ structure exhibited two non-equal sites for Eu^3+^ occupation, but one of them was a ч. Therefore, the observed transition reflects the influence of the larger site. The average Eu–O distance in the polyhedral structure of Sr_3_(PO_4_)_2_ was 2.7476 Å. In the Sr_3_Eu(PO_4_)_3_, only one site was observed, with an average Eu–O distance of approximately 2.6754 Å. This point was clearly demonstrated in previous studies [23,60], where it was shown that for the ^5^D_0_ → ^7^F_0_ transition, the Eu–O distance has a direct correlation with the wavelength of the transition. Hence, line A corresponds to the Eu^3+^ environment in the Sr_3_Eu(PO_4_)_3_, while line B corresponds to the Eu^3+^ in Sr_3_(PO_4_)_2_ (Figure 8b). 

Regarding the SrMn0.2Eu sample, the ^5^D_0_ → ^7^F_0_ transition can be described by four Gaussian components (Figure 8c). The increase in the number of components was attributed to the formation of the β-Ca_3_(PO_4_)_2_-type structure (Figure 8c). Fitting line A remains unchanged in terms of the maximum and position, indicating the Eu^3+^ oxygen environment in the Sr_3_Eu(PO_4_)_3_. Line B corresponds to the Eu^3+^ in the Sr_3_(PO_4_)_2_ host. Additional lines C and D correspond to the Eu^3+^ in the strontiowhitlockite [13]. Therefore, some polyhedra in the strontiowhitlockite and Sr_3_(PO_4_)_2_ hosts were approximately the same, which is why lines B and C have very closely centered maximum values. 

The observed ^5^D_0_ → ^7^F_0_ transitions for SrMn0.4Eu and SrMn0.6Eu can be accurately fitted by three Gaussian components, indicating that Eu^3+^ ions were mainly involved in the Sr_3_Eu(PO_4_)_3_- and strontiowhitlockite-type hosts. For the single-phased samples SrMn0.8Eu and SrMn1.0Eu, the ^5^D_0_ → ^7^F_0_ transition can be fitted by two Gaussian components. This suggests that there are two different environments for Eu^3+^ in the host [13], as is shown in Figure 2 for strontiowhitlockite with the presence of SrO_10_ and SrO_9_ sites. 

**Figure 9:**
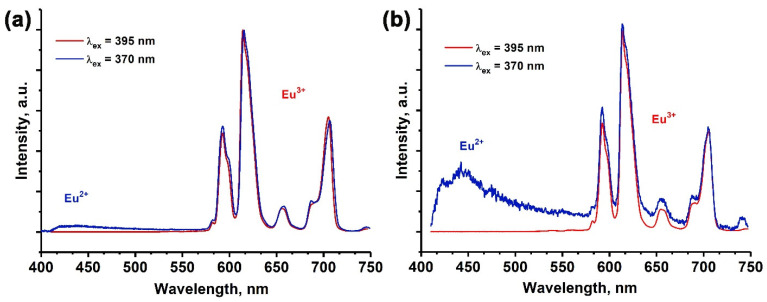
The PL spectra for Sr_9−*x*_Mn*_x_*Eu(PO_4_)_7_ with *x* = 0.2 (**a**) and 0.8 (**b**) (λ_exc_ = 395 and 370 nm).

According to the ESR data analysis, it was confirmed that Eu^2+^ ions were detected in the samples. As a result, the PL spectra were monitored for all the samples, employing an excitation wavelength of 370 nm (Figure 9). Notably, for the samples with *x* ≥ 0.2, distinct 4*f*5*d*–4*f* Eu^2+^ transitions were registered. The observed band appeared to be asymmetrical in shape and was predominantly centered around the wavelength of approximately 445 nm. 

The intensity of the Eu^2+^ emission band at ~445 nm was higher for the *x* = 0.8 sample (Figure 9b) compared to the *x* = 0.2 sample (Figure 9a). Consequently, it can be inferred that the addition of Mn^2+^ to the samples resulted in an overall increase in the total integral intensity of the Eu^2+^ transitions. The presence of Eu^2+^ emissions in the PL spectra indicates the abnormal reduction of Eu^3+^ ions in the strontiowhitlockite host. Moreover, the increase in the Mn^2+^ doping in the SrMn*x*Eu solid solutions led to an increase in the Eu^2+^ ion concentration and a more efficient reduction process. 

### 2.5. Temperature Dependence of Photoluminescence

**Figure 10:**
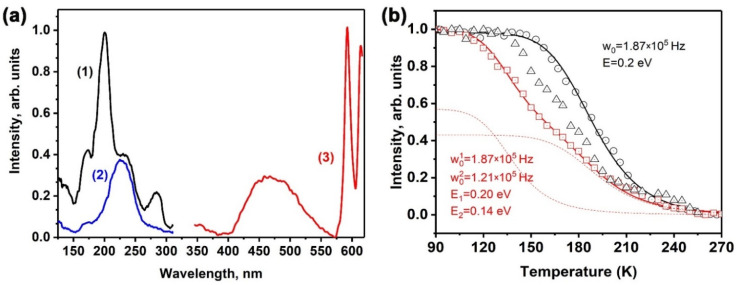
(**a**) The PLE spectra of (1) Eu^2+^ (λ_em_ = 470 nm) and (2) Eu^3+^ (λ_em_ = 595 nm), and (3) the PL spectrum (λ_exc_ = 230 nm for Sr_9.2_Mn0.8Eu(PO_4_)_7_ measured at 80 K); (**b**) the temperature dependence of Eu^2+^ luminescence in Sr_9−*x*_Mn*_x_*Eu(PO_4_)_7_ (*x* = 0.6 (black circles), *x* = 0.8 (black triangles), and *x* = 1.0 (red squares) with the two separate Mott–Seitz curves (dash red lines)).

Upon cooling to 80 K, a broad band appeared in the PL spectra centered at 470 nm (Figure 10a line 3). The PLE spectra of this band are shown in Figure 10a (Figure 10a lines 1 and 2). Under monitoring at 470 nm, the PLE spectrum consisted of several bands at 285, 230, 200, and 170 nm (Figure 10a line 1), with the most intense band being observed at 200 nm. When monitoring at 595 nm, the PLE spectrum showed only one band peaked at 230 nm, corresponding to charge transfer effects.

Figure 10b demonstrates the temperature dependence of the luminescence intensity of an emission band centered at 470 nm under 230 nm excitation. The observed behavior of the dependence follows Mott’s law, with an activation energy (*E*) of 0.2 eV and a frequency factor (*w*_0_) of 1.87∙10^5^ Hz. This luminescence band is potentially associated with 5*d*–4*f* transitions in the Eu^2+^ ions. Therefore, the activation energy in the temperature quenching curve of the luminescence could correspond to the energy difference between the high-energy excited 5*d* states of Eu^2+^ and the bottom of the conduction band.

With an increasing concentration of Mn^2+^ ions, the temperature dependence of luminescence underwent changes. At lower temperatures, the luminescence intensity of Eu^2+^ was observed to be quenched (Figure 10b). This phenomenon can be explained by the interaction between the Mn^2+^ ions and the surrounding environment. In the case of SrMn1.0Eu, the temperature dependence of the luminescence can be well described by the sum of two Mott’s functions, which provides valuable insights into the underlying mechanisms of luminescence:

(1) $$I\left(T\right)=0.43{\left(1+w_{0}^{1}\exp\left(\frac{{-E}_{1}}{k_{B}T}\right)\right)}^{-1}+0.57{\left(1+w_{0}^{2}\exp\left(\frac{{-E}_{2}}{k_{B}T}\right)\right)}^{-1}$$

where $w_{0}^{1}=1.87\times 10^{5}\;Hz$, $w_{0}^{2}=1.21\times 10^{5}\;Hz$, $E_{1}=0.20$ eV, and $E_{2}=0.14$ eV. It was observed that the samples with a high Mn^2+^ content exhibited the presence of two distinct Eu^2+^ centers, which provides evidence for the existence of multiple Eu^2+^ centers within the SrMn1.0Eu sample.

### 2.6. The Decay Curves

**Figure 11:**
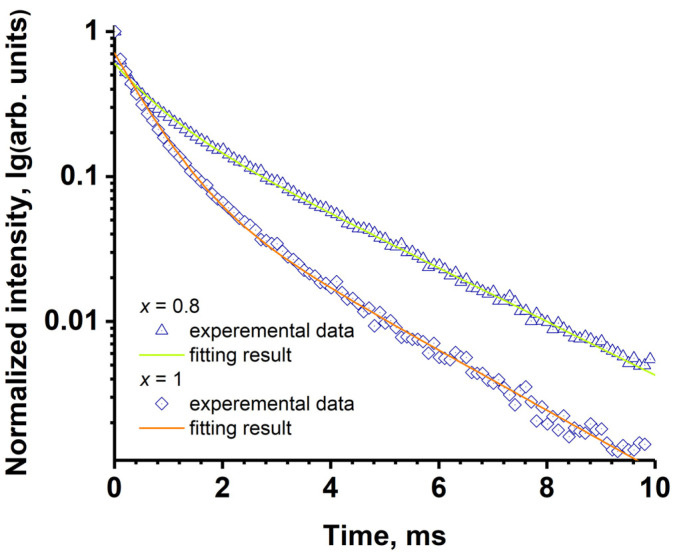
The decay curves for Sr_9−*x*_Mn*_x_*Eu(PO_4_)_7_ (*x* = 0.8, 1.0) samples monitored at 395 nm excitation and 615 nm emission.

The decay curves were collected for the single-phased samples of SrMn0.8Eu and SrMn1.0Eu (Figure 11). The curves were well fitted by the double exponent function:

*I*(*t*) = *A*_1·_exp(−*t*/τ_1_) + *A*_2·_exp(−*t*/τ_2_) (2)

where *I*(*t*) is the intensity at time *t*, τ_1_ and τ_2_ are the decay times for the exponential components, and *A*_1_ and *A*_2_ are fitting constants. The average lifetimes were calculated using the following equation [61]:

(3) $$\tau =\frac{A_{1}{\tau_{1}}^{2}+A_{2}{\tau_{2}}^{2}\;}{A_{1}\tau_{1}+A_{2}\tau_{2}}$$

The calculated average lifetime for the SrMn0.8Eu was 1.97 ms (*A*_1_ = 0.3, τ_1_ = 2.35, *A*_2_ = 0.3, τ_2_ = 0.69), while for the SrMn1.0Eu, it was 1.19 ms (*A*_1_ = 0.11, τ_1_ = 2.1, *A*_2_ = 0.6, τ_2_ = 0.59). The values were lower compared to other Eu^3+^-doped strontiowhitlockite samples [62]. The low decay time of the Eu^3+^ emission may indicate charge transfer processes from the Eu^3+^ levels. 

### 2.7. The Abnormal Reduction Process

The presence of Eu^2+^ ions in the host, as indicated by the ESR and photoluminescence data, suggests that the Eu^3+^ reduced to Eu^2+^ during the synthesis in air. This reduction process has been observed previously in several works [25,32,63,64,65,66]. The authors propose that this abnormal reduction of Eu^3+^ to Eu^2+^ in air occurs through a charge compensation mechanism. In [67], the conditions for the reduction process in solid-state compounds were proposed. A detailed analysis of previously obtained data on Eu^3+^ spectra in different phosphate hosts reveals this abnormal reduction, which follows Pei’s rules with one additional modification (Table 2). 

Due to the ionic radii mismatch between Sr^2+^ and Eu^3+^ ions, Sr-based phosphates with a β-TCP-type structure are suitable for reducing Eu^3+^ to Eu^2+^ in air. Additionally, the synthesis products (NH_3_, see Section 2.1, reaction) create a weak reducing atmosphere, further promoting the reduction of Eu^3+^ to Eu^2+^. One possible reduction scheme, based on diffuse absorption, is Mn^2+^ + Eu^3+^ ↔ Mn^3+^ + Eu^2+^, where Mn^2+^ acts as the reducing agent and Eu^3+^ as the oxidant. The reduction process occurs due to the susceptibility of the structure to the reducing agent NH_3_ and the presence of Mn^2+^ ions.

### 2.8. Color Characteristics

**Figure 12:**
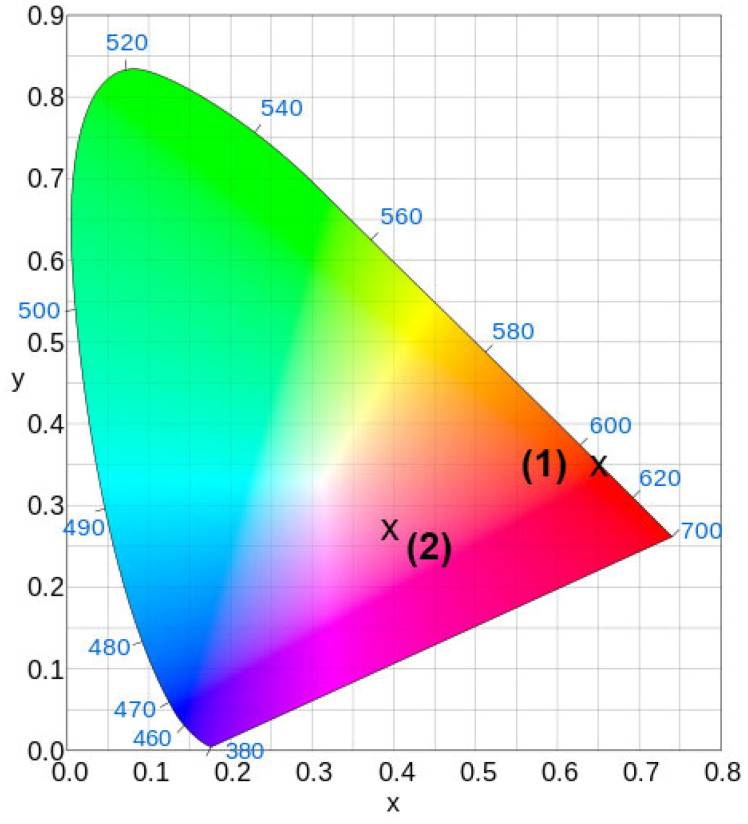
The CIE coordinates for Sr_9.2_Mn_0.8_Eu(PO_4_)_7_ at λ_exc_ = 395 (1) and 370 (2) nm.

One of the important characteristics of phosphors is their CIE coordinates. These coordinates can be determined from the emission spectral data of the samples. The calculated results are shown in Figure 12. For the Sr_8.2_Mn_0.8_Eu(PO_4_)_7_ sample monitored at 395 nm, the color coordinates (0.647; 0.351) fell in the red-orange region on the CIE diagram (Figure 12, point 1). When excited at 370 nm, the color coordinates (0.399; 0.270) were within the pink region (Figure 12, point 2). 

## 3. Materials and Methods

### 3.1. Synthesis 

The series of Sr_9−*x*_Mn*_x_*Eu(PO_4_)_7_ (named SrMn*x*Eu, *x* = 0, 0.2, 0.4, 0.6, 0.8, 1) was synthesized using a high-temperature solid-state method. Stoichiometric mixtures of NH_4_H_2_PO_4_ (99.9%), SrCO_3_ (99.9%), MnCO_3_ (99.99%), and Eu_2_O_3_ (99.9%) were used as the starting materials. The amounts of reactants were calculated based on the following reaction:

(18 − 2*x*) SrCO_3_ + 2*x* MnCO_3_ + 14 NH_4_H_2_PO_4_ + Eu_2_O_3_ → 2 Sr_9−*x*_Mn*_x_*Eu(PO_4_)_7_ + 18 CO_2_ + 14 NH_3_ +21 H_2_O

The required amounts were mixed in an agate mortar with acetone for better homogenization. The resulting mixture was then transferred to an alundum crucible for stepwise heating:

- Slow heating up to 200 °C for 8 h, followed by annealing for 8 h in air.
- Heating to 1100 °C for 12 h, followed by annealing for 24 h in air.

This slow heating method was chosen to guarantee the uniform removal of volatile by-products from the reaction. The powder X-ray diffraction (PXRD) patterns of the precursors were checked using the JCPDS PDF-2 database, which did not show any reflections of impurity phases.

### 3.2. Characterization

Powder X-ray diffraction (PXRD) patterns of SrMn*x*Eu were collected on a Thermo ARL X’TRA powder diffractometer (CuK*α* radiation, *λ* = 1.5418 Å, Bragg–Brentano geometry, Peltier-cooled CCD detector). The PXRD data were collected over the 5°–70° 2*θ* range with steps of 0.02°. The Le Bail decomposition [68] was applied for the PXRD analysis using the JANA2006 software [69]. 

The second harmonic generation (SHG) signal was measured with a *Q*-switched YAG: Nd laser at *λ*_ω_ =1064 nm in the reflection mode.

For collecting the electron spin resonance (ESR) spectra, the powder samples were placed in a quartz tube and measured using an RE–1306 X–band ESR spectrometer (KBST, Smolensk, Russia) operating at a frequency of 9.3841 GHz at room temperature.

The VUV excitation luminescence was recorded using an MDR-2 monochromator (LOMO, Saint Petersburg, Russia) equipped with a grate of 1200 lines per mm. A Hamamatsu photomodule operating in the photon counting mode was used for the detection. Excitation was carried out using a Hamamatsu L7293-50 deuterium lamp with a magnesium fluoride window coupled with a VMR-2 vacuum monochromator. The excitation spectra were corrected using sodium salicylate. The registration of the temperature was performed using a type K thermocouple.

The diffuse absorption spectra were registered with a Lambda 950 spectrophotometer equipped with integrated sphere in the transmittance regime (Perkin-Elmer, New-York city, NY, USA).

The photoluminescence emission (PL) and excitation (PLE) spectra were recorded under excitation in the UV-Vis spectral region using a specialized laboratory set-up. An ARC 150 W Xe lamp was used as an excitation source. The primary monochromator MDR-206 was used for the selection of the excitation wavelength. The PLE spectra were measured with a spectral resolution of 5 nm. The luminescence spectra were detected using an Oriel MS257 spectrograph using a 300 gr/mm or 2400 gr/mm grating with a spectral resolution of 1.5 nm and 0.32 nm, respectively (“low” and “high” resolution). A Marconi 30-11 CCD detector was used for the registration. The luminescence spectra were corrected for the spectral sensitivity of the registration channel. The measured excitation spectra were normalized on the excitation spectrum of yellow lumogen. All measurements were performed at room temperature.

The luminescence decay time was registered using a Perkin-Elmer LS-55 spectofluorimeter (Perkin-Elmer, New-York city, NY, USA) equipped with a Xe lamp with a 10 mks pulse duration. All measurements were performed at room temperature and corrected for the sensitivity of the spectrometer.

## 4. Conclusions

New solid solutions of Sr_9−*x*_Mn*_x_*Eu(PO_4_)_7_ with a strontiowhitlockite structure were synthesized using a high-temperature solid-state method in air. The concentration limit of Mn^2+^ ions in the host for the formation of the strontiowhitlockite (or β-TCP) phase was determined at *x* ≥ 0.8. The composition of the multi-phased samples was confirmed by X-ray and SHG analyses. The quenching of the Eu^3+^ emission was observed under a 395 nm excitation with the Mn^2+^ concentration. It was proposed that Eu^3+^ excitation was quenched through the Mn^2+^ levels, following potential reaction scheme Mn^2+^ + Eu^3+^ ↔ Mn^3+^ + Eu^2+^. The ESR and PL spectra measurements confirmed the abnormal partial reduction of Eu^3+^ → Eu^2+^ in air and the presence of Eu^2+^ ions in the host. Both 4*f*5*d*–4*f* Eu^2+^ and 4*f*–4*f* Eu^3+^ transitions were observed in the PL spectra under a 370 nm excitation. The intensity of the Eu^2+^ emission decreased with heating from 80 K to 270 K in Sr_9−*x*_Mn*_x_*Eu(PO_4_)_7_. The detailed analysis of the ^5^D_0_ → ^7^F_0_ transition showed the presence of several non-equal Eu^3+^ environments. The decay curves were measured, and it was found that the decay times for the Eu^3+^ levels were lower compared to other strontiowhitlockite-based phosphors. The conditions for the occurrence of Eu^3+^ → Eu^2+^ reduction in air were discussed.

## Figures and Tables

**Table 1:** The phase composition and the SHG signals for Sr_9−*x*_Mn*_x_*Eu(PO_4_)_7_ samples.

	Whitlockite-Type	Palmierite-Type	Eulytite-Type	SHG
	Sr_9_Fe_1.5_(PO_4_)_7_	Sr_3_(PO_4_)_2_	Sr_3_Eu(PO_4_)_3_	
	sp.gr. $R\bar{3}$*m*	sp.gr. $R\bar{3}$*m*	sp.gr. $I\bar{4}$3*d*	
	Centrosymmetric	Centrosymmetric	Non-Centrosymmetric	
*x* = 0	0	45%	55%	1.1
*x* = 0.2	49%	23%	28%	0.7 ± 0.1
*x* = 0.4	67%	13%	20%	0.5 ± 0.1
*x* = 0.6	83%	0	17%	0.3 ± 0.1
*x* = 0.8	100%	0	0	0.1 ± 0.1
*x* = 1.0	100%	0	0	0.1 ± 0.1

**Table 2:** The conditions for the reduction of Eu^3+^ to Eu^2+^ in air.

Conditions	Present Work	Remarks
(1) No oxidizing ions should be present in the host.	There were no oxidizing ions in the SrMn*x*Eu host.	
(2) The dopant *R*^3+^ ions must replace host cations with a different oxidation state.	Eu^3+^ replaced Sr^2+^ ions in SrMn*x*Eu host.	In the β-Ca_3_(PO_4_)_2_ structure, Eu^3+^ can also replace Ca^2+^ ions in the host. Eu^2+^ emission was found in some hosts.
(3) The host cations must have similar radii to the divalent *R*^2+^ ions.	*r*_VIII_(Eu^2+^) = 1.25 Å was close to *r*_VIII_(Sr^2+^) = 1.26 Å.	The similarity of the ionic radii explains the more common abnormal reduction in air in the Sr-based host compared to the Ca-based one.

## Data Availability

The data presented in this study are available on request from the corresponding author.
